# Precision Switching and Coupled Motion in a [3]Rotaxane Molecular Machine

**DOI:** 10.1002/anie.6152447

**Published:** 2026-06-30

**Authors:** Leonardo Andreoni, Jessica Groppi, Alberto Credi, Serena Silvi

**Affiliations:** ^1^ Dipartimento di Chimica Industriale “Toso Montanari” Alma Mater Studiorum ‐ Università di Bologna Bologna Italy; ^2^ CLAN‐Center for Light Activated Nanostructures Alma Mater Studiorum ‐ Università di Bologna and National Research Council of Italy Bologna Italy; ^3^ Institute for Organic Synthesis and Photoreactivity (ISOF) National Research Council of Italy (CNR) Bologna Italy; ^4^ Dipartimento di Chimica “G. Ciamician” Alma Mater Studiorum ‐ Università di Bologna Bologna Italy

**Keywords:** ammonium, bypiridinium, crown ethers, molecular machines, rotaxanes, triazolium

## Abstract

We report the synthesis and the characterization of a multicomponent molecular machine based on a [3]rotaxane architecture. The system is composed by two crown ether macrocycles and an axle with three recognition sites for the rings: ammonium (AmH^+^), bipyridinium (Bpy^2+^), and triazolium (Trz^+^). The position of the two rings can be precisely controlled *via* a sequence of chemical and electrochemical inputs: the two rings can be located on neighboring stations, forced on the same station or separated at the opposite extremities of the axle. This complex mechanism is elucidated by a combination of NMR spectroscopy and voltammetric techniques, allowing to characterize the thermodynamics of the reaction network. The investigation shows that each ring is influenced by the presence and position of the other, resulting in a coupled motion, a critical feature for the development of next‐generation molecular machines.

## Graphical abstract

A [3]rotaxane, composed by two crown ether macrocycles and an axle presenting three recognition sites, is operated through both chemical and electrochemical stimuli. The position of the macrocycles is precisely controlled by sequential stimulation and the processes are fully reversible. The characterization of the system revealed the macrocycles affect each other relative affinities for the stations, resulting in the coupling of their motion.

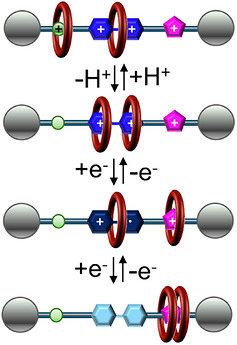

## 1 Introduction

The study of artificial molecular machines is a vibrant research field that experienced a rapid growth over the last three decades [1, 2, 3, 4, 5]. Interest in this field stems from the desire to emulate biomolecular machines, that are ubiquitous in living organisms and fundamental in many life‐sustaining processes [6, 7], as well as to explore the potential of artificial analogues to bring about innovative applications in technology and medicine [8, 9, 10, 11]. However, in contrast to their biological counterparts, artificial molecular devices are generally characterized by simple structures often based on mechanically interlocked molecules [12, 13]. Within this class of compounds, [2]rotaxanes are the most common architecture, while higher order rotaxanes are much less investigated, most likely due to the increased complexity associated with their synthesis and characterization [14]. However, the higher number of interlocked components can lead to emerging properties like sequence isomerism [15, 16], enhanced photochemical properties [17, 18, 19] and complex movements, such as ring‐through‐ring shuttling [20], and folding/unfolding motions [21]. Although these features make higher order rotaxanes promising candidates for the development of new generations of molecular machines, examples of such systems are relatively rare [18, 21, 22, 23, 24, 25, 26, 27, 28, 29, 30, 31, 32, 33], and, besides a couple of exceptions [30, 31], they are all based on palindrome or symmetric structures. Such structures, while synthetically more accessible, prevent a precise control over the rings movement, as a single stimulus triggers indiscriminately the motion of both rings.

When considering multi‐component systems (i.e., comprising three or more components), the interactions between the different parts and the coupling of their motion are key for their operation as molecular machines. This feature is pivotal in biomolecular machines, in which the motion of one component influences the neighboring units, allowing the accomplishment of complex tasks. One can consider as an example the “hand‐over‐hand” coupled motion of the kinesin walking along the microtubules [34]. The studies of multi‐component artificial devices that show interaction or coupling between the different sub‐components are somewhat limited to date [28, 30, 35, 36].

**FIGURE 1:**
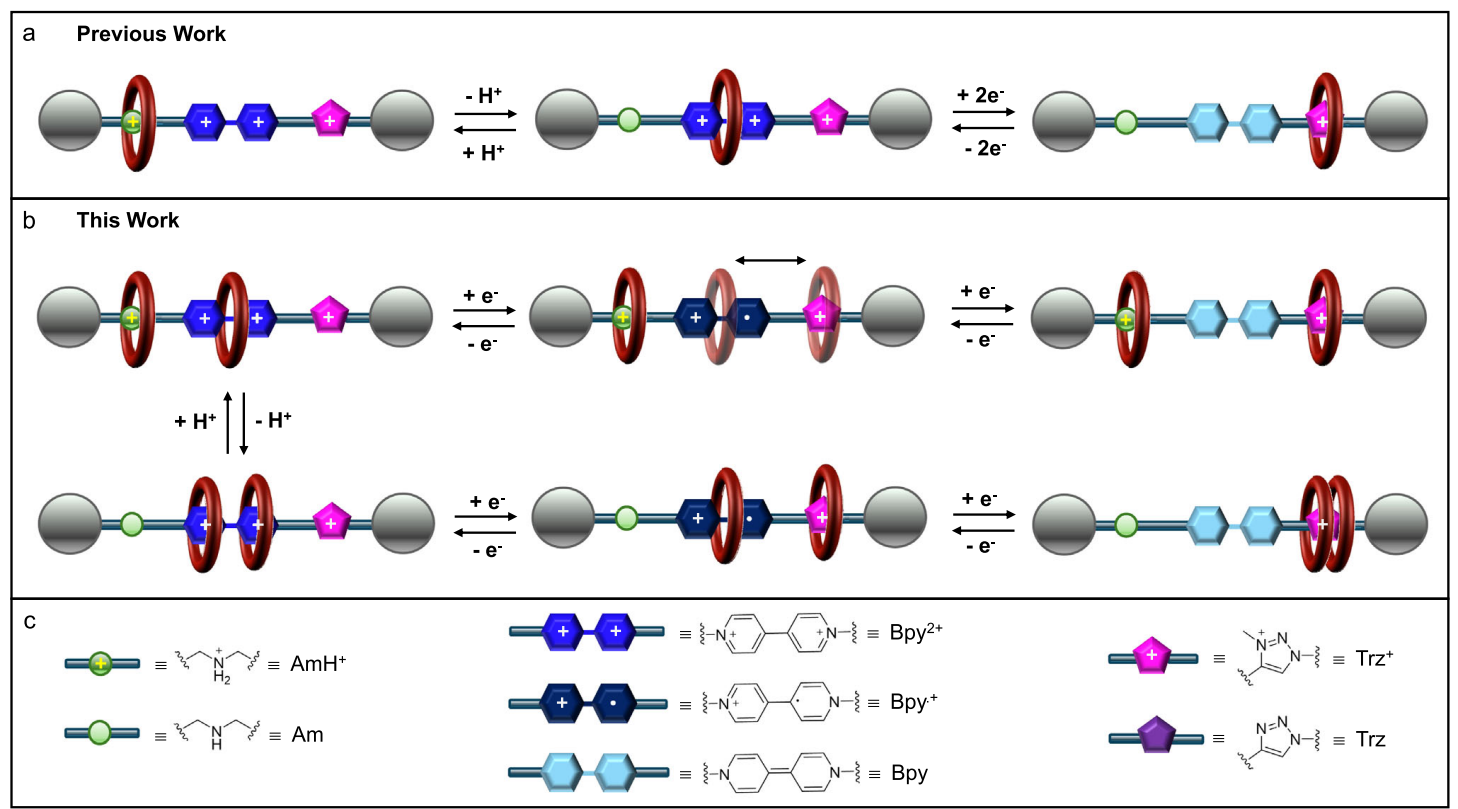
Schematic representation of the switching patterns in (a) a previously investigated three station [2]rotaxane and (b) the present three‐station [3]rotaxane. Panel (c) shows the structure formulas and cartoon representations of the main subunits in the rotaxane axle and their abbreviations as recalled in the text.

We recently described a three‐station [2]rotaxane in which the translocation of the ring can be governed by means of orthogonal stimuli (Figure 1a) [37]. This system is composed by a dibenzo‐24‐crown‐8 (DB24C8) macrocycle and an axle‐like molecule with three recognition sites: ammonium (AmH^+^, primary station), bipyridinium (Bpy^2+^, secondary station) and triazolium (Trz^+^, tertiary station). AmH^+^ interacts with the ring through hydrogen bonding and it can be switched‐off by deprotonation [38, 39, 40]. Bpy^2+^ establishes charge‐transfer interactions with the aromatic units of the ring [41, 42], and its electron‐accepting properties can be frustrated by electrochemical reduction [43], as it displays two reversible reduction processes to the radical cation and the neutral form [44]. Altogether, the addition of base induces the motion of the ring from the primary to the secondary station, while the reduction of the Bpy^2+^ induces the motion to the tertiary station. The whole process is reversible and a reverse sequence of inputs allows to reset the system.

Here, we report an example of multi‐component molecular machine in which the linear motion of two macrocycles along an axle is coupled and can be precisely controlled by applying sequential reversible stimuli. We discuss the synthesis and characterization of a novel [3]rotaxane based on the same molecular components as the parent [2]rotaxane [37], but presenting two identical crown ethers surrounding the axle. In analogy with the [2]rotaxane, the interaction between the macrocycles and the recognition sites can be modulated *via* chemical and electrochemical stimuli (Figure 1b); however, the presence of two rings leads to a significantly more complex switching pattern. Indeed, the two DB24C8 macrocycles can be induced to share the same station, surround adjacent stations or be separated, depending on the combination and sequence of stimuli. Remarkably, the motion of each ring depends on the position of the other, resulting in the coupling of the two components.

## 2 Results and Discussion

### 2.1 Synthesis and Model Compounds

**FIGURE 2:**
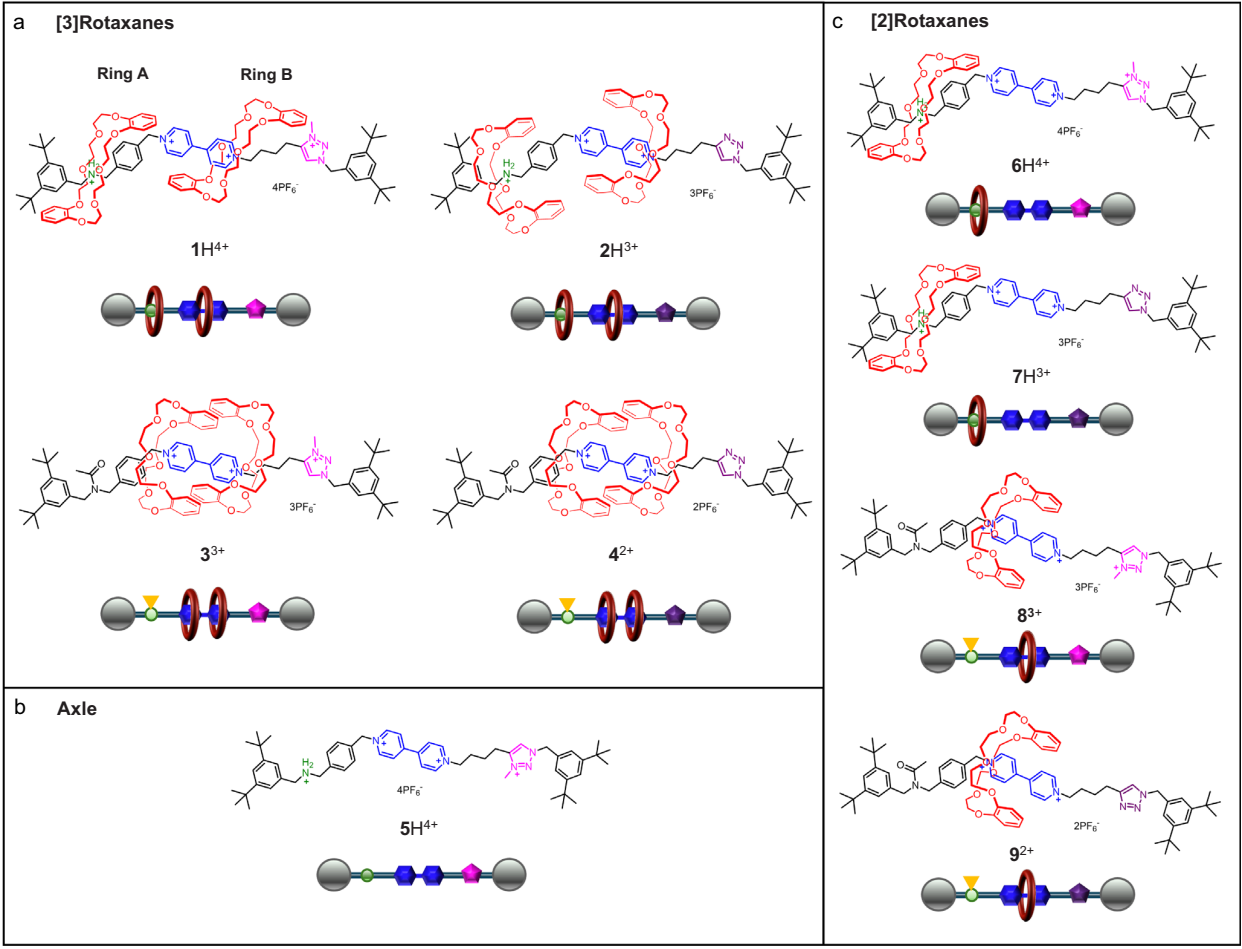
Chemical structure and cartoon representation of (a) compound **1**H^4+^ and its [3]rotaxane models **2**H^3+^, **3** ^3+^, and **4** ^2+^, (b) free axle **5**H^4+^, and (c) [2]rotaxane models **6**H^4+^, **7**H^3+^, **8** ^3+^, and **9** ^2+^.

The synthetic approach adopted to prepare [3]rotaxane **1**H^4+^ (Figure 2a and Scheme S1) is similar to that previously reported for the synthesis of [2]rotaxane **6**H^4+^ (Figure 2c). The open axle **10**H^3+^, presenting both AmH^+^ and Bpy^2+^, was first obtained, followed by complexation with DB24C8 in dichloromethane (DCM): this step is crucial to get to the [3]rotaxane rather than the [2]rotaxane compound. To drive the formation of the desired product, the crown ether had to be present in excess (3 equivalents) and at a high concentration (0.3 M) in order to favor the threading of a second ring to reside on the Bpy^2+^ station, characterized by a much lower association constant with DB24C8 compared to AmH^+^ [45]. The assembled [3]pseudorotaxane could then be locked by means of a copper (I) catalyzed alkyne‐azide click reaction (CuAAC) with 3,5‐di‐t‐butylbenzylazide to obtain precursor **2**H^3+^. Methylation of the triazole with iodomethane allowed to install the third recognition site Trz^+^. Derivatives **3** ^3+^ and **4** ^2+^ were obtained by deprotonation of AmH^+^ with resin‐supported diazabicycloundecene (DBU) base and acetylation with acetic anhydride.

Notably, 24C8 crown ethers, despite their flexibility, have a too small cavity to allow ring‐through‐ring shuttling in a compound like **1**H^4+^ [20]. Hence, the two macrocycles will retain their reciprocal position during operation and can be differentiated according to their position with respect to the non‐symmetrical axle. The DB24C8 closer to AmH^+^ will be henceforth identified as ring A, while the one closer to Trz^+^ will be labelled ring B.

A complex system such as **1**H^4+^, which combines two macrocycles and multiple recognition sites switchable through orthogonal stimuli, could potentially access a high number of states. Therefore, several model compounds had to be designed, prepared and examined to understand and characterize its operation. Among the [3]rotaxane structures (Figure 2a), **2**H^3+^ and **3** ^3+^ are bi‐stable rotaxanes missing the tertiary Trz^+^ and primary AmH^+^ station, respectively. In these model compounds the rings can either distribute between the two recognition sites available or they can both reside at the station for which they present the highest affinity. **4** ^2+^ is a rotaxane where the only available station for the two macrocycles is Bpy^2+^. The axle **5**H^4+^ (Figure 2b) is a reference for the free stations, while compounds **6**H^4+^, **7**H^3+^, **8** ^3+^, and **9** ^2+^ (Figure 2c) are the [2]rotaxane structures corresponding to **1**H^4+^, **2**H^3+^, **3** ^3+^, and **4** ^2+^, respectively, and provide information on the behavior of the system when only one DB24C8 is present [37].

### 2.2 NMR Characterization

**FIGURE 3:**
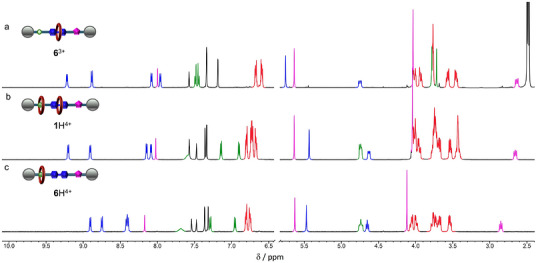
Partial ^1^H NMR spectra (500 MHz, CD_3_CN, 298 K) of (a) [2]rotaxane **6** ^3+^, (b) [3]rotaxane **1**H^4+^, and (c) [2]rotaxane **6**H^3+^; color coding of the signals indicates: Bpy^2+^ – blue; Trz^+^–pink; AmH^+^ – green; DB24C8 – red.

The formation of the [3]rotaxanes was confirmed by NMR spectroscopy and mass spectrometry (Sections S2 and S3). The stack of ^1^H NMR spectra reported in Figure 3 illustrates the chemical shifts of relevant protons in **1**H^4+^ (Figure 3b), model compound **6**H^4+^ (Figure 3c) and its deprotonated derivative **6** ^3+^ (Figure 3a) in CD_3_CN. The comparison shows that **1**H^4+^ is a [3]rotaxane in which the two rings encircle, respectively, AmH^+^ and Bpy^2+^. In particular, the match between the chemical shift of the methylene protons next to the ammonium moiety in **1**H^4+^ and **6**H^4+^ (*δ∼*4.7 ppm, green) demonstrates that AmH^+^ is located within the cavity of a crown ether. At the same time, the comparison of the chemical shifts of the protons in **1**H^4+^ with those in **6** ^3+^, where deprotonation induced the translation of the macrocycle to the bipyridinium recognition site [37], indicate that one of the rings must be surrounding Bpy^2+^ (*δ*∼8.0–9.5 ppm, blue).

**FIGURE 4:**
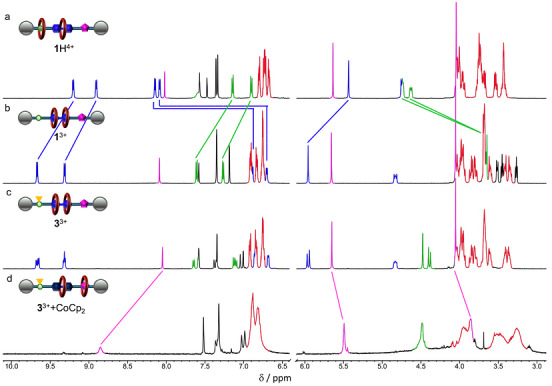
Partial ^1^H NMR spectra (500 MHz, CD_3_CN, 298 K) of (a) [3]rotaxane **1**H^4+^, (b) **1**H^4+^ after the addition of 1 eq of DBU base to obtain **1** ^3+^; (c) **3** ^3+^; (d) **3** ^3+^ after the addition of 2 eq of CoCp_2_; color coding of the signals indicates: Bpy^2+^–blue; Trz^+^–pink; AmH^+^–green; DB24C8 – red.

^1^H NMR spectroscopy also allowed to monitor the movement of the macrocycles upon sequential deactivation of AmH^+^ and Bpy^2+^ in CD_3_CN (Figure 4). The deprotonation of **1**H^4+^ (Figure 4a) with ∼1 equivalent of 1,8‐diazabicyclo[5.4.0]undec‐7‐ene (DBU, p*K*_a_ = 24.3) [46] to generate **1** ^3+^ caused the transfer of ring A onto Bpy^2+^, already occupied by ring B, as revealed by a significant shift of the signals associated with the bipyridinium protons (Figure 4b, blue). In particular, while the doublets corresponding to the protons adjacent to the positively charged nitrogen move to lower fields, as they become surrounded by the H‐bond accepting chains of the macrocycles, the second set of doublets is shifted by about 1.5 ppm to higher field, due to the shielding by the aromatic groups of both DB24C8 rings [47, 48]. An analogous pattern was observed when the AmH^+^ is fully deactivated by acetylation in **3** ^3+^ (Figure 4c) and in **4** ^2+^, where the only active recognition site is Bpy^2+^ (Figure S26). Notably, despite the presence of Trz^+^ as an available station, the two macrocycles preferentially share the Bpy^2+^ unit both in **1** ^3+^ and **3** ^3+^, instead of localizing one macrocycle on each station. This observation confirms that triazolium represents a very weak station compared to bipyridinium. Despite 1D NMR spectra showing that both rings A and B are located on Bpy^2+^, no cross peaks clearly indicating interactions between them were present on the 2D ROESY spectra of rotaxanes **3** ^3+^ and **4** ^2+^ (Figures S17 and S23). Nonetheless, to investigate whether the two crown ethers affect each other's interaction with Bpy^2+^, the apparent acidity constant of AmH^+^ in [3]rotaxane **2**H^3+^ was determined via ^1^H NMR titration with quinuclidine (QQ, p*K*_a_ = 19.5) [46] in CD_3_CN, obtaining p*K*_a_ = 18.3 (Section S5). In rotaxanes of this kind, it is well established that the stronger the interaction between the ring and the AmH^+^ station, the lower the apparent acidity of the latter, and this phenomenon is counterbalanced by the presence of an alternative station for the ring [49, 50]. Thus, the p*K*_a_ of **2**H^3+^ can be correlated to the AmH^+^/Bpy^2+^ co‐conformation equilibrium constant (Section S6): a comparison with the p*K*_a_ of [2]rotaxane **7**H^3+^ (p*K*_a_ = 17.2) [37] suggests that in the [3]rotaxane the presence of ring B at Bpy^2+^ lowers by a 10‐fold factor the affinity of the latter for ring A, giving rise to an anti‐cooperative effect.

The deactivation of Bpy^2+^ by reduction was performed using cobaltocene (CoCp_2_) as a chemical reductant. In order to avoid degradation of **1** ^3+^ [51], the reaction was performed on model compound **3** ^3+^. According to the electrochemical characterization of **3** ^3+^ (vide infra), the reduction potentials of Bpy^2+^ are *E*_1/2_(Bpy^2+^/Bpy^+∙^) = –0.67 V vs. SCE and *E*_1/2_(Bpy^+∙^/Bpy^0^) = –0.99 V vs. SCE (Table 1); hence, when **3** ^3+^ is treated with CoCp_2_ (*E*_1/2_ = –0.94 V vs. SCE) [52], it can be envisioned that only the reduction to the radical cation Bpy^+∙^ can be achieved quantitatively. In these conditions, the ^1^H NMR spectrum (Figure 4d) shows a drastic downfield shift of the Trz^+^ protons (in pink), which indicates that at least one of the macrocycles (ring B) is encircling this station, as previously reported for model compound **7** ^3+^ (Figure S28) [37].

**TABLE 1:** Reduction potential values (V vs. SCE) of the examined compounds in Ar purged CH_3_CN at 298 K.^a^

Compounds	*E*_1_	*E*_2_	*E*_3_ ^b^
**1**H^4+^	−0.49	−0.81	−1.89
**1** ^3+^	−0.68	−0.99	−2.01
**2**H^3+^	−0.53	−0.99	—
**2** ^2+^	−0.78	−1.14	—
**3** ^3+^	−0.67	−0.99	−2.07
**4** ^2+^	−0.78	−1.21	—
**5**H^4+^ ^c^	−0.36	−0.78	−1.71
**6**H^4+^ ^c^	−0.35	−0.77	−1.70
**6** ^3+^ ^c^	−0.51	−0.83	−1.92
**7**H^3+^ ^c^	−0.36	−0.79	—
**7** ^2+^ ^c^	−0.56	−1.00	—
**8** ^3+^ ^c^	−0.50	−0.83	−1.91
**9** ^2+^ ^c^	−0.55	−1.01	—

^a^ ^)^Halfwave potential values taken from CV, unless noted otherwise.

^b^ ^)^Peak potential of the poorly reversible process, taken from DPV.

^c^ ^)^Potential values reported in Ref. [37].

### 2.3 Electrochemical Characterization

The electrochemical characterization of **1**H^4+^ and its model compounds was performed by both cyclic voltammetry (CV) and differential pulse voltammetry (DPV) in CH_3_CN and CH_2_Cl_2_ (Section S7). As the behavior of **1**H^4+^ in the two solvents is similar, the discussion will focus on the experiments performed in CH_3_CN. It is known that Bpy^2+^ undergoes two monoelectronic and reversible reduction processes, whereas Trz^+^ sustains a poorly reversible reduction one [37]. Moreover, when the two stations are surrounded by one or more macrocycles, their reduction potentials are shifted toward negative values [37, 38]. Finally, the oxidation state of bipyridinium can affect its affinity for crown ethers [37]. Thus, electrochemical inputs play a dual role: (a) provide information on the state of the system—that is, on the position of the macrocycle(s)—–and (b) modify it by modulating the interactions between stations and macrocycle(s).

Table 1 summarizes the reduction potential values recorded for all compounds in CH_3_CN: *E*_1_ and *E*_2_ are the potential values associated with the two reduction processes of Bpy^2+^, while *E*_3_ refers to the reduction potential of Trz^+^.

The results obtained on the [2]rotaxanes are pivotal to elucidate the switching mechanism of **1**H^4+^ and are hereafter summarized: (i) the reduction potential values of Bpy^2+^ in the axle are not affected by the state of the other two stations (ammonium/amide/amine, triazolium/triazole); (ii) when the rotaxane is protonated and the ring resides on the AmH^+^ station, the reduction potential values of Bpy^2+^ and Trz^+^ correspond to the ones of their free counterparts; (iii) when Bpy^2+^ is encircled by one ring, the first reduction process is shifted to more negative potential values; as for the second reduction of Bpy^2+^, (iv) in the absence of Trz^+^, also this process is shifted (∼200 mV) to more negative values, whereas (v) in presence of Trz^+^ it is only slightly shifted (∼50 mV), but the irreversible reduction of Trz^+^ occurs at more negative values. Overall, these results indicate that, upon deprotonation, the ring shuttles from the deactivated primary station to the secondary Bpy^2+^ station; upon subsequent two‐electron reduction of the Bpy^2+^ unit, the ring shuttles to the tertiary Trz^+^ station.

**FIGURE 5:**
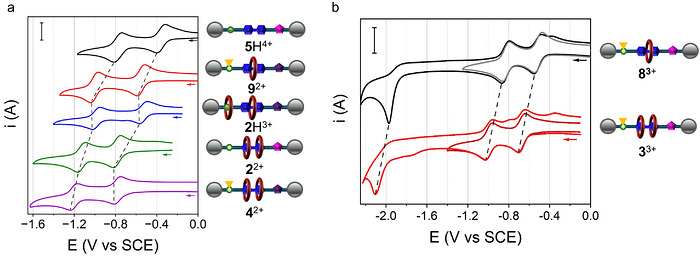
Cyclic voltammograms (Ar purged CH_3_CN, 298 K, TEAPF_6_ 100 equivalents, scan rate: 100 mV s^−1^) of: (a) **5**H^4+^ (3.8 × 10^−4^ M, black line, scalebar: 8 µA), **9** ^2+^ (3.5 × 10^−4^ M, red line, scalebar: 6 µA), **2**H^3+^ (2.9 × 10^−4^ M, blue line, scalebar: 5 µA), **2** ^2+^ (2.9 × 10^−4^ M, green line, scalebar: 5 µA), **4** ^2+^ (3.4 × 10^−4^ M, purple line, scalebar: 5 µA); (b) **8** ^3+^ (3.5 × 10^−4^ M, black line, scalebar: 8 µA), **3** ^3+^ (2.4 × 10^−4^ M, red line, scalebar: 8 µA); since new anodic peaks appear after the reduction of Trz^+^, both the full scan CV and the CV where the scan is reversed before Trz^+^ reduction are reported.

The comparison of the CVs recorded for free axle **5**H^4+^ (black), [2]rotaxane **9** ^2+^ (red) and [3]rotaxane **4** ^2+^ (purple), shown in Figure 5a, clearly demonstrates how the reduction potential of Bpy^2+^ is affected by an increasing number of crown ethers surrounding it. Indeed, both *E*_1_ and *E*_2_ of **4** ^2+^, where both rings are located on Bpy^2+^, are negatively shifted by more than 400 mV compared to the free axle, and by more than 200 mV with respect to **9** ^2+^ in which Bpy^2+^ is encircled by one ring.

Figure 5a also compares the CVs of **2**H^3+^ (blue) and **9** ^2+^ (red): the electrochemical properties of the [3]rotaxane are analogous to those of the [2]rotaxane where one macrocycle surrounds Bpy^2+^. Such an observation confirms that in **2**H^3+^ ring A encircles AmH^+^, while ring B surrounds Bpy^2+^, in line with the NMR data. The addition of 1 equivalent of the P_1_‐*t*Bu base (p*K*_a_ = 26.9) [53] to **2**H^3+^ causes the deprotonation of AmH^+^, affording **2** ^2+^ in which the primary station is de‐activated. In these conditions, the only available station for the rings is the bipyridinium one: in fact, the reduction potential values of **2** ^2+^ are similar to those of **4** ^2+^, suggesting that ring A has moved following deprotonation and is sharing Bpy^2+^ with ring B (Figures 1b and 5a) [54].

Finally, the comparison of the CVs of **8** ^3+^ (black) and **3** ^3+^ (red) (Figure 5b) allowed to understand how the two crown ethers behave in the [3]rotaxane upon reduction of Bpy^2+^ when only Trz^+^ is active. As determined by ^1^H NMR, in **3** ^3+^ both macrocycles surround Bpy^2+^: indeed, the first reduction peak is negatively shifted compared to the [2]rotaxane where only one ring encircles Bpy^2+^ (**8** ^3+^). The second reduction peak of **3** ^3+^ falls at *E*_2_ = –0.99 V, a value close to *E*_2_ = –1.01 V found for [2]rotaxane **9** ^2+^. This observation suggests that upon monoelectronic reduction of the Bpy^2+^ unit of **3** ^3+^, ring A stays around the Bpy^+∙^ radical cation, while NMR data demonstrate that ring B moves to Trz^+^ (Figures 1b and 4d). The complete reduction of bipyridinium to its neutral form Bpy^0^ causes a negative shift of the reduction potential of Trz^+^ (*E*_3_), indicating that the station is located in the macrocycle cavity [37]. Notably, the negative shift in the [3]rotaxane is larger than that determined for the [2]rotaxane (*E*_3_ = –2.07 and –1.91 V, respectively; Table 1), strongly suggesting that both macrocycles interact with Trz^+^ upon two‐electron reduction of Bpy^2+^.

**SCHEME 1:**
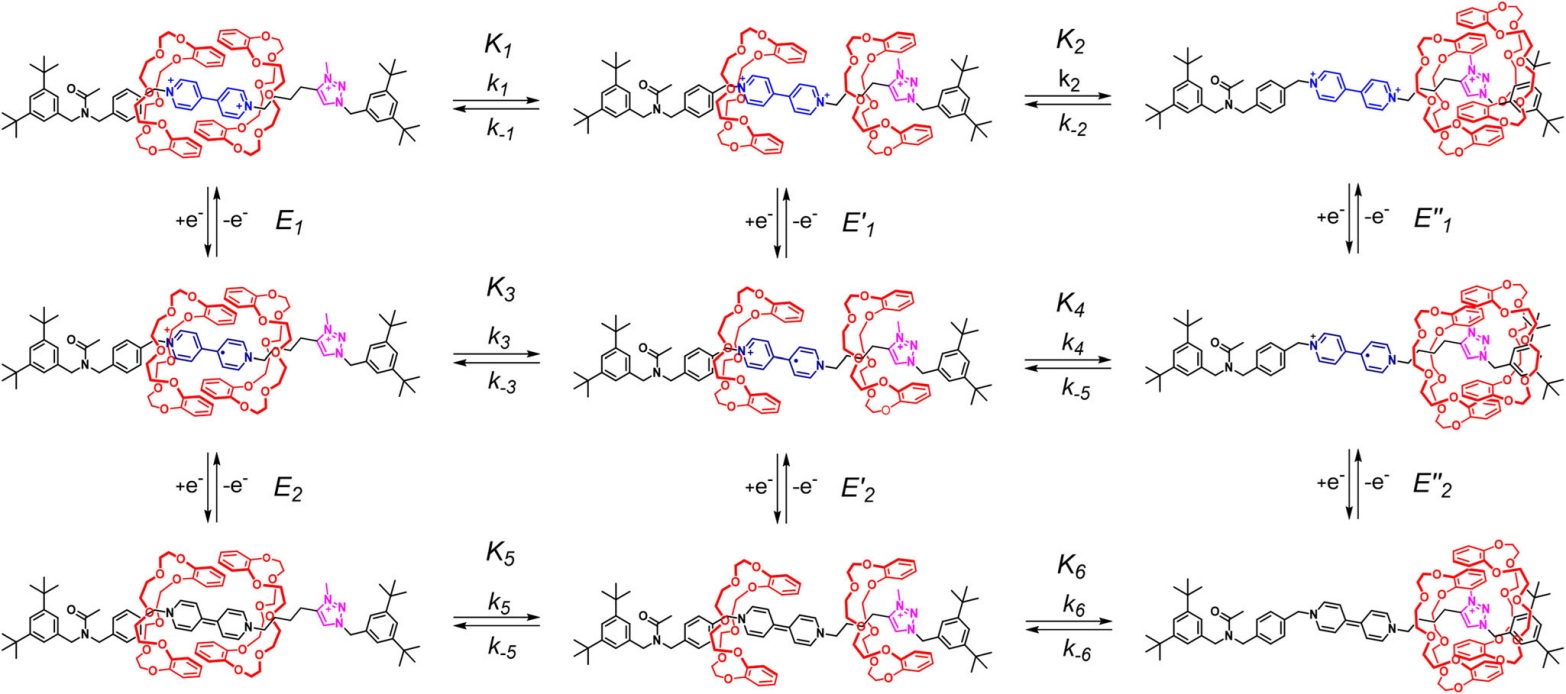
Co‐conformational equilibria of **3** ^3+^ for the different redox states of Bpy^2+^. The vertical processes represent the redox reactions of Bpy^2+^, whereas the horizontal processes represent the shuttling of DB24C8 between Bpy^2+^ and Trz^+^. Counterions are omitted for the sake of clarity.

The experimental CVs of **3** ^3+^ were fitted to curves simulated according to the reaction network represented in Scheme 1, with the aim of determining the distribution of the species in the co‐conformational equilibria of the [3]rotaxane for the different redox states of Bpy^2+^ (Section 7.2).

The equilibrium constants values calculated from the fitting are reported in Table 2. The results confirmed the experimental observations just described for **3** ^3+^, namely: (i) in the initial state both rings surround Bpy^2+^ (*K*_1_<< 1); (ii) after the first Bpy^2+^ reduction, ring B moves to Trz^+^ (*K*_3_>> 1), while ring A remains on Bpy^+∙^ (*K*_4_<< 1); (iii) when bipyridinium is fully reduced to Bpy^0^ in rotaxane **3** ^+^ a fraction of rings A shuttles toward Trz^+^. It was estimated that, for **3** ^+^ in CH_2_Cl_2_, approximately 82% (*K*_6_ = 4.7) of the [3]rotaxanes present two rings surrounding the triazolium, whereas in CH_3_CN this occurs in about 63% (*K*_6_ = 1.7) of the [3]rotaxanes. Despite the fitting suggesting that in CH_3_CN nearly 40% of the **3** ^+^ species should still have ring A located at Bpy^0^, there is no experimental evidence of such co‐conformation in the voltammograms, as the reduction of Trz^+^ surrounded by only one ring (around –1.9 V vs. SCE, see Table 1) was not observed. Nevertheless, the complexity of the model used to fit the voltammetries implies that the fitting predictions, albeit qualitatively accurate, may fail to precisely capture the distribution of the rings when the affinity difference for the available stations is so small.

**TABLE 2:** Computed thermodynamic constants of the co‐conformational equilibria, related to the different oxidation states of Bpy^2+^, in **3** ^3+^ (Scheme 1), obtained by fitting of the cyclic voltammetries in CH_3_CN and CH_2_Cl_2_ at 298 K.

	CH_3_CN	CH_2_Cl_2_
*K*_1_	(1.1 ± 0.2) × 10^−2^	(5 ± 1) × 10^−3^
*K*_2_	(1.3 ± 0.4) × 10^−7^	(5 ± 1) × 10^−8^
*K*_3_	(9 ± 2) × 10^1^	(8 ± 2) × 10^1^
*K*_4_	(3.2 ± 0.9) × 10^−4^	(6 ± 1) × 10^−4^
*K*_5_	(1.4 ± 0.3) × 10^4^	(2.8 ± 0.6) × 10^4^
*K*_6_	1.7 ± 0.5	4.7 ± 0.9

Overall, cyclic voltammetric experiments and fittings indicate that the first reduction of the bipyridinium induces the translocation of ring B on Trz^+^, while the second reduction induces the motion of ring A on the same station, at least in the majority of the [3]rotaxane species in a population. The whole mechanism is reversible, as highlighted by the excellent reversibility of the Bpy^2+^ redox processes. Therefore, the first re‐oxidation of the bipyridinium causes the backward shuttling of ring A on Bpy^+∙^, while the second re‐oxidation triggers the shuttling of ring B on the restored Bpy^2+^ (Figure 1b).

**FIGURE 6:**
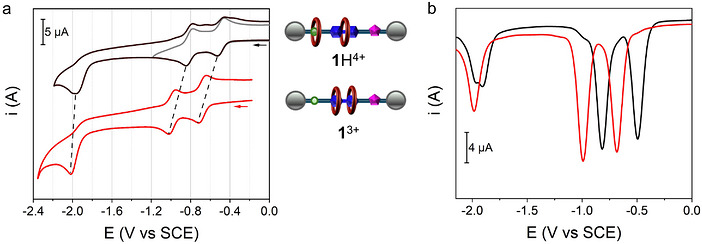
Voltammetric curves (Ar purged CH_3_CN, 298 K, TEAPF_6_ 100 equivalents) recorded for a solution of **1**H^4+^ (2.7 × 10^−4^ M, black and gray lines), and after the addition of 1 equivalent of P_1_‐*t*Bu base to obtain **1** ^3+^(red line). (a) Cyclic voltammograms (scan rate: 50 mV s^−1^); since new anodic peaks appear after the reduction of Trz^+^, both the full scan CV and the CV where the scan is reversed before Trz^+^ reduction are reported. (b) Differential pulse voltammetries (scan rate: 20 mV s^−1^, pulse height: 75 mV).

With this information in hand, the operation of three‐station [3]rotaxane **1**H^4+^ can now be analyzed from the electrochemical point of view. Figure 6 reports the comparison of the CV (Figure 6a) and DPV (Figure 6b) traces associated with **1**H^4+^ (black) and its deprotonated form **1** ^3+^ (red). In the protonated state, the Bpy^2+^ reduction potentials are analogous to those of the [2]rotaxane **8** ^3+^ (Table 1), in which Bpy^2+^ reduction causes the translocation of the ring to Trz^+^. This observation is consistent with ring A surrounding AmH^+^ and ring B initially surrounding Bpy^2+^, as also demonstrated by the NMR data, and suggests that after full reduction of the bipyridinium unit, ring B moves onto Trz^+^ while ring A maintains its starting position.

The voltammetric traces of **1**H^4+^ exhibit two partially overlapped reduction processes at –1.89 and –1.98 V vs. SCE, related to Trz^+^ (Figure 6). The first one corresponds to the protonated [3]rotaxane and denotes that Trz^+^ is surrounded by one macrocycle. The second one can be ascribed to the presence of **1** ^3+^ generated by partial deprotonation of AmH^+^ triggered by the reduction of Trz^+^, as previously reported for **6**H^4+^ (see Section S7.1) [37].

Upon addition of 1 equivalent of P_1_‐*t*Bu base, AmH^+^ is deprotonated, thus generating **1** ^3+^ in which the primary station is de‐activated. In analogy with the peak potentials recorded for two‐station [3]rotaxane **3** ^3+^, all the processes become negatively shifted (Table 1) [55]. Therefore, it can be concluded that after deprotonation ring A shares Bpy^2+^ with ring B, in line with the NMR results. Moreover, upon consecutive electrochemical reductions of the bipyridinium, a sequential shuttling of rings B and A toward the triazolium site occurs, which is ultimately surrounded by both rings.

Overall, **1**H^4+^ can be switched with a combination of both chemical and electrochemical stimuli between six different states, as depicted in Figure 1. The deprotonation of AmH^+^, followed by two reductions of Bpy^2+^ allows to induce the stepwise sequential directional motion of the two crown ethers along the axle, in a similar fashion as the progressive motion observed for a single ring in [2]rotaxane **6**H^3+^ [37]. It should be noted, however, that while reduction prior to deprotonation has no consequence on the position of the ring in the [2]rotaxane, it is not the same for the [3]rotaxane, where the reduction of Bpy^2+^ affects the position of ring B.

On the one hand, the similarity of operation between the two rotaxanes highlights the modular nature of this family of compounds, in which the addition of new components introduces new features, while maintaining the ones of the simpler system. Such a behavior is useful from a design viewpoint because it allows a great deal of predictability. On the other hand, it is worth noting that the new properties introduced in the derived species do not simply add to those of the parent species. In fact, the simultaneous presence of two rings and their relative position on the axle affects not only the motion dynamics but also each other's affinity for the stations, as shown by the apparent p*K*_a_ of AmH^+^ in the [3]rotaxane, which is higher than that of the [2]rotaxane. Moreover, while in **1**H^4+^ the displacement of ring B from Bpy^2+^ to Trz^+^ is accomplished by two‐electron reduction of Bpy^2+^, as deduced by comparison with the electrochemical behavior of [2]rotaxane **8** ^3+^, in the deprotonated **1** ^3+^, it can be inferred from the characterization of model compound **3** ^3+^ that the same process is observed after one‐electron reduction of Bpy^2+^. Such an effect can be ascribed to the presence of a second macrocycle on Bpy^2+^ that lowers the overall affinity of DB24C8 for the station, analogously to the result obtained for the deprotonation of AmH^+^. In other terms, the two macrocycles influence each other's affinity for the recognition sites in an anti‐cooperative manner which drives them in a sequential and directionally controlled motion along the axle.

## 3 Conclusion

The NMR and electrochemical investigation, allowed to elucidate the switching behavior of **1**H^4+^. In this multi‐stable [3]rotaxane, the movement of the rings can be controlled precisely through the combination of one chemical and two electrochemical inputs (Figure 1): the two rings can be induced to share the same station, to reside in neighboring stations or to be located in two stations at the opposite extremities of the axle. The [3]rotaxane is a complex system but not a complicated one, as the right combination of stimuli allows to quantitatively switch it to a given state, among the six available ones.

The current system represents an unprecedented example of control over the motion of the rings in higher‐order rotaxanes. These architectures are relatively poorly explored for the development of molecular machines and most of the examples are based on palindrome structures [18, 21, 22, 23, 24, 25, 26, 27, 28, 29, 30, 32, 33], which do not allow to individually trigger the motion of the rings. Moreover, there are very few examples of systems in which the motion of the rings is coupled [28, 30], a feature which holds great potential for the emergence of advanced functions, as shown for [3]catenanes molecular motors [35, 36]. **1**H^4+^ shows both features (i.e., addressability of the rings and coupling of their motion), and the three stations are positioned in a sequential way along the axle, leading to a directional movement of the two rings.

Altogether, this study shows how, starting from simple and well‐known building blocks, the increase of components in artificial molecular machines results in the emergence of novel properties. We believe that this strategy will be key to drive the current state of the art in the field of artificial molecular machines toward the complexity shown in natural systems.

## Author Contributions

**Leonardo Andreoni**: investigation, writing – review and editing, and formal analysis. **Jessica Groppi**: investigation, writing – original draft, writing – review and editing, and formal analysis. **Alberto Credi**: conceptualization, funding acquisition, writing – review and editing, and supervision. **Serena Silvi**: supervision, writing – original draft, and writing – review and editing.

## Conflicts of Interest

The authors declare no conflicts of interest.

## Supporting information



The authors have cited additional references within the Supporting Information [37, 40, 46, 52, 56, 57, 58, 59].
**Supporting file**: anie73233‐sup‐0001‐SuppMat.docx.

## Data Availability

The data that support the findings of this study are available from the corresponding author upon reasonable request.
