# Supplementary material for: Precision Switching and Coupled Motion in a [3]Rotaxane Molecular Machine
Source: Angew Chem Int Ed Engl. 2026 Jun 30;65(34):e6152447. doi: 10.1002/anie.6152447 (PMC13480727; doi:10.1002/anie.6152447)
Supplement: Supplementary file 1 [file ANIE-65-e6152447-s001.docx]

**Precision Switching and Coupled Motion in a [3]Rotaxane Molecular Machine**

Leonardo Andreoni,^[a,b]^ Jessica Groppi,^*[b,c]^ Alberto Credi,^[a,b]^ and Serena Silvi^*[b,d]^

[a]	Dr. Leonardo Andreoni, Prof. Alberto Credi
Dipartimento di Chimica Industriale “Toso Montanari”
Università di Bologna,
Via P. Gobetti 85, 40129, Bologna, Italy

[b]	Dr. Leonardo Andreoni, Dr. Jessica Groppi, Prof. Alberto Credi, Prof. Serena Silvi
CLAN-Center for Light Activated Nanostructures,
Istituto ISOF-CNR,
Via P. Gobetti 101, 40129 Bologna, Italy
Email: [serena.silvi@unibo.it](mailto:serena.silvi@unibo.it), [jessica.groppi@cnr.it](mailto:jessica.groppi@cnr.it)

[c]	Dr. Jessica Groppi,
Institute for Organic Synthesis and Photoreactivity (ISOF), National Research Council of Italy (CNR),
Via P. Gobetti 101, 40129 Bologna, Italy.

[d]	Prof. Serena Silvi
Dipartimento di Chimica “G. Ciamician”,
Università di Bologna,
Via P. Gobetti 85, 40129, Bologna, Italy.

**SUPPORTING INFORMATION**

**Table of contents**

[1.	Materials and methods	2](#_Toc224829817)

[2.	Synthetic procedures	4](#_Toc224829818)

[3.	NMR Spectra	10](#_Toc224829819)

[4.	^1^H NMR Monitoring of DB24C8 translation to Trz^+^ induced by chemical reduction of Bpy^2+^	32](#_Toc224829820)

[5.	p*K*_a_ Determination *via* ^1^H NMR spectroscopy experiments	34](#_Toc224829821)

[6.	Relation between apparent acidity constant and co-conformational
 equilibrium constants of [3]rotaxane 2H^3+^	36](#_Toc224829822)

[7.	Electrochemical measurements	38](#_Toc224829823)

[7.1 Cyclic Voltammetries and Differential Pulse Voltammetries	38](#_Toc224829824)

[7.2 Cyclic voltammetries fitting	44](#_Toc224829825)

[7.3 Relation between reduction potential of Trz^+^ and co-conformation
 equilibrium constants of [3]rotaxane **1**^3+^	46](#_Toc224829826)

[References	49](#_Toc224829827)

# 1. **Materials and methods**

**General.** Model compounds **5**H^4+^, **6**H^4+^, **7**^3+^**, 8**^2+^ and **9**^2+^ as well as precursors **10**H^3+^ and 3,5-Di-tert-butylbenzylazide were synthesized according to literature procedures.^[^^[1]]^ All reagents and chemicals were purchased from Merck, VWR international or Fluorochem and used as received unless otherwise stated. Solvents were dried according to literature procedures. Thin layer chromatography was performed on TLC Silica gel 60 F254 coated aluminium plates from Merck. Flash column chromatography was performed using Merck Silica 40 (230-400 mesh size or 40-63 mm) as the stationary phase. Size exclusion chromatography was performed using Biorad Biobeads SX-1 as the stationary phase and dichloromethane as the eluent.

**NMR Spectroscopy.** NMR spectra were recorded on an Agilent DD2 spectrometer operating at 500 MHz. Chemical shifts are quoted in part per million (ppm) relative to tetramethylsilane using the residual solvent peak as a reference standard and all coupling constants (*J*) are expressed in Hertz (Hz).

**Mass Spectrometry.** High resolution mass spectrometry (HRMS) measurements were performed on a Waters Xevo G2-XS instrument equipped with an ESI source and a Q-TOF ion analyzer.

**Electrochemical Measurements.** Cyclic voltammetric (CV) and differential pulse voltammetric (DPV) experiments were carried out in argon-purged CH_2_Cl_2_ and CH_3_CN (Sigma-Aldrich) with an Autolab 30 multipurpose instrument interfaced to a PC. The working electrode was a glassy carbon electrode (Amel, 0.07 cm^2^), carefully polished with an alumina-water slurry on a felt surface, immediately before use. The counter electrode was a Pt wire, separated from the solution by a frit, an Ag wire was employed as a quasi-reference electrode and ferrocene was present as an internal standard (*E*_1/2_= –0.46 V *vs* SCE in CH_2_Cl_2_; *E*_1/2_= –0.395 V *vs* SCE in CH_3_CN). The concentration of the examined compounds was ranging from 0.2 to 0.5 mM. Tetrabutylammonium hexafluorophosphate (TBAPF_6_) and tetraethylammonium hexafluorophosphate (TEAPF_6_) were added as supporting electrolyte in CH_2_Cl_2_ and CH_3_CN respectively, in a 100-fold proportion with respect to the sample concentration. Cyclic voltammograms were obtained at scan rates varying from 20 to 3000 mV s^−1^. Differential pulse voltammetries were performed with a scan rate of 20 mV s^−1^ (pulse height 75 mV). The IR compensation was used and every effort was made throughout the experiments in order to minimize the resistance of the solution. The electrochemical reversibility of the voltammetric wave of ferrocene was taken as an indicator of the absence of uncompensated resistance effects. For reversible processes the halfwave potential values were obtained from the average of the cathodic and anodic cyclic voltammetric waves. The potential values for poorly reversible processes were estimated from the DPV peaks. The uncertainty on the equilibrium constants determined from digital simulations of the voltammetric curves is the standard deviation obtained from the fitting and was estimated to be at least ±20%.

# 2. **Synthetic procedures**

Scheme S1 - Synthetic steps to compounds **1**H^4+^, **2**H^3+^, **3**^3+^, **4**^2+^. Conditions: a) DB24C8, CH_2_Cl_2_, r.t., 2 hour; b) 3,5-di-tert-butylbenzylazide, Cu(CH_3_CN)_4_PF_6_, CH_2_Cl_2_, N_2_, r.t., 48 hours; c) Resin supported DBU, DCM, r.t., 40 min; d) Acetic anhydride, CH_2_Cl_2_, r.t., 48 hours; e) NH_4_PF_6_, MeOH; f) CH_3_I, CH_2_Cl_2_, r.t., 3 days.

**[3]Rotaxane 2H**^3+^

Compound **10**H^3+^ (0.2 g, 0.2 mmol) was dissolved in anhydrous DCM (5 ml) and DB24C8 (0.3, 0.6 mmol) was added. The solution was stirred for 2 hours to allow for the formation of the complex. Di-*tert*-butylbenzylazide (0.16 g, 0.6 mmol) was dissolved in anhydrous DCM (1.5 ml) and added to the stirring solution. The mixture was bubbled with nitrogen for 10 minutes, followed by the addition of Cu(CH_3_CN)_4_PF_6_ (0.08 g, 0.2 mmol). The solution was stirred at room temperature for 48 hours under nitrogen atmosphere. The reaction solvent was removed and the product was purified over silica column (eluent: CHCl_3_ then CHCl_3_:MeOH = 9.6:0.4 then CHCl_3_:MeOH = 7:3) followed by size exclusion chromatography. The product was a light yellow solid obtained in 20% yield (0.1 g). **^1^H NMR (500 MHz, CD_2_Cl_2_, 298 K)** δ= 9.44 (d, *J* = 6.5, 2H, H_13_), 8.57 (d, *J* = 6.5, 2H, H_10_), 7.74 (d, *J* = 6.5, 2H, H_11_), 7.70 (bs, 2H, H_5_), 7.62 (d, *J* = 6.5, 2H, H_12_), 7.43 (s, 1H, H_1_), 7.42 (s, 1H, H_21_), 7.32 (s, 2H, H_3_), 7.29 (d, *J* = 8.0, 2H, H_7_), 7.26 (s, 1H, H_18_), 7.13 (s, 2H, H_20_), 6.99 (d, *J* = 8.0, 2H, H_8_), 6.86-6.74 (m, 8H, H_ArA_), 6.67-6.61 (m, 8H, H_ArB_), 5.43 (s, 4H, H_9_+H_19_), 4.89 (t, *J* = 8.7, 2H, H_14_), 4.73-4.6 (m, 4H, H_4_+H_6_), 4.14-4.10 (m, 4H, H_αA_), 4.05-4.00 (m, 8H, H_αA_+ H_αB_), 3.89-3.73 (m, 20H, H_βA_+ H_αB_+ H_βB_), 3.69-3.65 (m, 4H, H_γA_), 3.64-3.57 (m, 8H, H_25_ H_γB_), 3.53-3.49 (m, 4H, H_γA_), 2.70 (t, *J* = 8.0, 2H, H_17_), 2.36-2.29 (m, 2H, H_15_), 1.77-1.71 (m, 2H, H_16_), 1.29 (s, 18H, H_22_), 1.22 (s, 18H, H_2_). **^13^C NMR (125 MHz, CD_2_Cl_2_, 298 K)** δ= 152.1, 151.5, 148.0, 147.9, 147.6, 147.5, 144.9, 134.8, 133.9, 132.3, 131.5, 130.8, 129.1, 127.6, 123.95, 123.9, 123.5, 123.1, 122.8, 121.8, 121.6, 121.1, 112.8, 112.4, 71.1, 71.0, 70.6, 70.3, 68.4, 68.1, 64.6, 61.7, 54.9, 52.4, 35.2, 35.1, 31.5, 31.4, 29.2, 27.2, 26,0. **HRMS (ESI+):** m/z calc. for C_102_H_137_N_6_O_16_PF_6_^2+^ [M]^2+^: 924.9961, found: 924.9944.

**[3]Rotaxane 4**^2+^

Rotaxane **2**H^3+^ (50 mg, 23 μmol) was dissolved in DCM (5 ml) and resin bound diazabicycloundecene (DBU) base (5 mg) was added. The mixture was stirred for 40 minutes at room temperature to allow for deprotonation of the compound. Resin bound DBU was filtered off and the solvent removed. The crude obtained was checked by ^1^H NMR to verify deprotonation (95%). The solid was then dissolved in DCM (5 ml) and acetic anhydride (3 ml) was added. The solution was stirred at room temperature for 48 hours. The reaction solvent and excess anhydride were removed under vacuum. The solid obtained was dissolved in MeOH and a saturated solution of NH_4_PF_6_ in MeOH was added to exchange the anion; the solvent volume was reduced and water was added to precipitate the product, which was filtered and dried under vacuum. The product was purified by size exclusion chromatography. The product was a dark yellow solid obtained in 60% yield (30 mg). **^1^H NMR (500 MHz, CD_2_Cl_2_, 298 K)** δ= 9.66 (m, 2H, H_10-minor isom_ +H_10-major isom_), 9.35 (d, *J* = 6.6, 2H, H_13_), 7.63 (d, *J* = 8.0, 2H, H_8-minor isom_), 7.55 (d, *J* = 8.0, 2H, H8_-major isom._), 7.42 (s, 1H, H_21_), 7.34 (s, 1H, H_1-major isom._), 7.32 (s, 1H, H_1-minor isom._), 7-21-7.12 (m, 7H, H_11_+ H_12_+H_18_+H_20_), 7.02 (d, *J* = 8.0, 2H, H_7-major isom._), 6.97-6-96 (m, 4H, H_7-minor isom._+H_3-minor isom._), 6.92 (s, 2H, H_3-major isom._), 6.84-6.75 (m, 8H, H_ArB_), 6.65-6.55 (m, 8H, H_ArA_), 6.08 (s, 2H, H_9-minor isom._), 6.03 (s, 2H, H_9-major isom._), 5.42 (s, 2H, H_19_), 5.01-4.96 (m, 2H, H_14_), 4.45 (s, 2H, H_4-minor isom._), 4.39 (s, 2H, H_4-major isom._), 4.36 (s, 2H, H_6-major isom._), 4.23 (s, 2H, H_6-minor isom._), 4.10-3.98 (m, 12H, H_αA_+ H_αB_), 3.91-3.85 (m, 8H, H_αB_+ H_βB_), 3.78-3.63 (m, 20H, H_βA_+H_βB_+H_γB_), 3.44-3.30 (m, 8H, H_γA_), 2.74-2.69 (m, 2H, H_17_), 2.42-2.36 (m, 2H, H_15_), 2.14 (s, 3H, H_5-major isom._), 2.11 (s, 3H, H_5-minor isom._), 1.80-1.73 (m, 2H, H_16_), 1.29 (s, 18H, H_21_), 1.28 (s, 18H, H_2_). **^13^C NMR (125 MHz, CD_2_Cl_2_, 298 K)** δ= 171.1, 170.8, 152.1, 151.9, 151.5, 149.2, 149.1, 148.8, 148.6, 148.5, 148.2,147.9, 147.8, 139.4, 138.3, 136.8, 136.0, 134.8, 132.0, 131.9, 131.8, 131.3, 128.2, 126.8, 123.9, 123.8, 123.2, 123.0, 122.7, 122.5, 121.9, 121.7, 121.5, 121.2, 121.0, 112.5, 112.3, 112.2, 71.6, 71.2, 70.6, 70.4, 68.7, 68.6, 65.1, 63.9, 63.8, 61.6, 54.8, 52.3, 51.0, 48.8, 48.4, 35.1, 35.0, 31.5, 30.0, 29.1, 27.4, 26.1, 22.1, 21.9. **HRMS (ESI+):** m/z calc. for C_104_H_140_N_6_O_17_PF_6_^+^ [M]^+^: 1890.9950 found: 1890.9917.

**[3]Rotaxane 1H**^4+^

Rotaxane **2**H^3+^ (100 mg, 45 μmol) was dissolved in CH_3_I (5 ml) and the solution was left stirring at room temperature for 72 hours. The excess CH_3_I was removed under vacuum and the crude was dissolved in MeOH. A saturated solution of NH_4_PF_6_ in MeOH was added to exchange the anion. The solvent volume was reduced and H_2_O was added to precipitate the product, which was then filtered and dried under vacuum. The product was purified by size exclusion chromatography to obtain a light-yellow solid in 85% yield (90 mg). **^1^H NMR (500 MHz, CD_2_Cl_2_, 298 K)** δ= 9.29 (d, *J* = 6.0, 2H, H_13_), 8.75 (d, *J* = 6.0, 2H, H_10_), 8.04 (d, *J* = 6.0, 2H, H_11_), 8.00 (s, 1H, H_18_), 7.93 (d, *J* = 6.0, 2H, H_12_), 7.65 (bs, 2H, H_5_), 7.53 (s, 1H, H_22_), 7.43 (s, 1H, H_1_), 7.32 (s, 2H, H_22_), 7.31 (s, 2H, H_3_), 7.21 (d, *J* = 8.0, 2H, H_7_), 6.99 (d, *J* = 8.0, 2H, H_8_), 6.85-6.73 (m, 8H, H_ArA_), 6.68 (bs, 8H, H_ArB_), 5.59 (s, 2H, H_20_), 5.45 (s, 2H, H_9_), 4.78 (t, *J* = 8.0, 2H, H_14_), 4.71-4.64 (m, 4H, H_4_+H_6_), 4.12-4.07 (m, 7H, H_19_+H_αA_), 4.01-3.93 (m, 12H, H_αA_+ H_αB_), 3.79-3.2 (m, 16H, H_βA_+ H_βB_), 3.69-3.66 (m, 4H, H_γA_), 3.56-3.42 (m, 12H, H_γA_+ H_γB_), 2.64 (t, *J* = 8.0, 2H, H_17_), 2.27-2.21 (m, 2H, H_15_), 1.67-1.58 (m, 2H, H_16_), 1.33 (s, 18H, H_23_), 1.21 (s, 18H, H_2_). **^13^C NMR (125 MHz, CD_2_Cl_2_, 298 K)** δ= 152.9, 152.1, 151.7, 149.4, 147.7, 147.6, 147.1, 145.2, 145.1, 144.7, 133.7, 132.5, 131.5, 130.7, 130.6, 129.5, 127.8, 127.7, 124.9, 124.7, 124.1, 124.0, 123.9, 121.8, 121.6, 112.8, 112.4, 71.2, 71.0, 70.7, 70.4, 68.3, 68.2, 64.5, 61.1, 58.7, 52.4, 37.8, 35.3, 35.2, 31.4, 29.2, 24.1, 23.3 . **HRMS (ESI+):** m/z calc. for C_103_H_140_N_6_O_16_P_2_F_12_^2+^ [M]^2+^: 1004.9899, found: 1004.9883.

**[3]Rotaxane 3**^3+^

Rotaxane **1**H^4+^ (50 mg, 22 μmol) was dissolved in DCM (5 ml) and resin bound diazabicycloundecene (DBU) base (5 mg) was added. The mixture was stirred for 40 minutes at room temperature to allow for deprotonation of the compound. Resin bound DBU was filtered off and the solvent removed. The solid was then dissolved in DCM (3 ml) and acetic anhydride (2 ml) was added. The solution was stirred at room temperature for 48 hours. The reaction solvent and excess anhydride were removed under vacuum. The solid obtained was dissolved in MeOH and a saturated solution of NH_4_PF_6_ in MeOH was added to exchange the anion; the solvent volume was reduced and water was added to precipitate the product, which was filtered and dried under vacuum. The product was purified by size exclusion chromatography. The product was a yellow solid obtained in 80% yield (40 mg). **^1^H NMR (500 MHz, CD_2_Cl_2_, 298 K)** δ= 9.73-9.70 (m, 2H, H_10_), 9.41-9.39 (m, 2H, H_13_), 7.83 (s, 1H, H_18._), 7.65 (d, *J* = 8.0, 2H, H_8-minor isom._), 7.57-7.55 (m, 3H, H_8-major isom.+_H_22_), 7.40-7.31 (m, 7H, H_1_+H_11_+H_12_+H_21_), 7.05 (d, *J* = 7.9, 2H, H_7-major isom._), 7.01 (d, *J* = 7.8, 2H, H_7-minor isom._), 7.02 (s, 2H, H_3-minor isom._), 7.00 (s, 2H, H_3-major isom._), 6.84-6.75 (m, 8H, H_ArA_), 6.72-6.61 (m, 8H, H_ArB_), 6.10 (s, 2H, H_9-minor isom._), 6.05 (s, 2H, H_9-major isom._), 5.57 (s, 2H, H_20_), 5.01-4.96 (m, 4H, H_14_), 4.46 (s, 2H, H_4-minor isom._), 4.40 (s, 2H, H_4-major isom._), 4.38 (s, 2H, H_6-major isom._), 4.26 (s, 2H, H_6-minor isom._), 4.08-3.99 (m, 19H, H_19_+ H_αA_+ H_αB_), 3.84-3.83 (m, 8H, H_βB_), 3.73-3.56 (m, 16H, H_βA_ + H_γB_), 3.44-3.31 (m, 8H, H_γA_), 2.62-2.57 (m, 2H, H_17_), 2.45-2.37 (m, 2H, H_15_), 2.14 (s, 3H, H_5-major isom._), 2.12 (s, 3H, H_5-minor isom._), 1.68-1.62 (m, 2H, H_16_), 1.34 (s, 18H, H_23_), 1.28 (s, 18H, H_2_). **^13^C NMR (125 MHz, CD_2_Cl_2_, 298 K)** δ= 171.1, 170.8, 152.9, 151.9, 151.5, 149.1, 149.0, 148.9, 148.8, 148.7, 147.9, 147.7, 145.0, 139.5, 138.4, 136.8, 136.0, 132.0, 131.9, 131.7, 131.3, 130.6, 128.3, 127.6, 126.8, 124.7, 124.1, 123.8, 123.7, 122.5, 121.9, 121.5, 121.4, 121.0, 112.4, 112.3, 71.6, 71.4, 70.6, 70.4, 68.7, 68.5, 64.0, 61.0, 58.6, 52.4, 48.9, 48.4, 37.7, 35.3, 35.1, 35.0, 31.6, 31.5, 31.4, 28.8, 24.3, 23.3, 22.1, 21.9. **HRMS (ESI+):** m/z calc. for C_105_H_141_N_6_O_17_PF_6_^2+^ [M]^2+^: 953.0076, found: 953.0092.

# 3. **NMR Spectra**

Figure S1 - ^1^H NMR spectrum of [3]rotaxane **1**H^4+^ (500 MHz, CD_2_Cl_2_, 298 K).

Figure S2 - ^13^C NMR spectrum of [3]rotaxane **1**H^4+^ (125 MHz, CD_2_Cl_2_, 298 K).

Figure S3 - ^1^H-^1^H COSY spectrum of [3]rotaxane **1**H^4+^ (500 MHz, CD_2_Cl_2_, 298 K).

Figure S4 - ^1^H-^1^H TOCSY spectrum of [3]rotaxane **1**H^4+^ (500 MHz, CD_2_Cl_2_, 298 K).

Figure S5 - ^1^H-^1^H ROESY spectrum of [3]rotaxane **1**H^4+^ (500 MHz, CD_2_Cl_2_, 298 K).

Figure S6 - ^1^H -^13^C HSQC spectrum of [3]rotaxane **1**H^4+^ (500 MHz, CD_2_Cl_2_, 298 K).

Figure S7 - ^1^H NMR spectrum of [3]rotaxane **2**H^3+^ (500 MHz, CD_2_Cl_2_, 298 K).

Figure S8 - ^13^C NMR spectrum of [3]rotaxane **2**H^3+^ (125 MHz, CD_2_Cl_2_, 298 K).

Figure S9 - ^1^H-^1^H COSY spectrum of [3]rotaxane **2**H^3+^ (500 MHz, CD_2_Cl_2_, 298 K).

Figure S10 - ^1^H-^1^H TOCSY spectrum of [3]rotaxane **2**H^3+^ (500 MHz, CD_2_Cl_2_, 298 K).

Figure S11 - ^1^H-^1^H ROESY spectrum of [3]rotaxane **2**H^3+^ (500 MHz, CD_2_Cl_2_, 298 K).

Figure S12 - ^1^H -^13^C HSQC spectrum of [3]rotaxane **2**H^3+^ (500 MHz, CD_2_Cl_2_, 298 K).

Figure S13 - ^1^H NMR spectrum of [3]rotaxane **3**^3+^ (500 MHz, CD_2_Cl_2_, 298 K).

Figure S14 - ^13^C NMR spectrum of [3]rotaxane **3**^3+^ (125 MHz, CD_2_Cl_2_, 298 K).

Figure S15 - ^1^H-^1^H COSY spectrum of [3]rotaxane **3**^3+^ (500 MHz, CD_2_Cl_2_, 298 K).

Figure S16 - ^1^H-^1^H TOCSY spectrum of [3]rotaxane **3**^3+^ (500 MHz, CD_2_Cl_2_, 298 K).

Figure S17 - ^1^H-^1^H ROESY spectrum of [3]rotaxane **3**^3+^ (500 MHz, CD_2_Cl_2_, 298 K).

Figure S18 - ^1^H -^13^C HSQC spectrum of [3]rotaxane **3**^3+^ (500 MHz, CD_2_Cl_2_, 298 K).

Figure S19 - ^1^H NMR spectrum of [3]rotaxane **4**^2+^ (500 MHz, CD_2_Cl_2_, 298 K).

Figure S20 - ^13^C NMR spectrum of [3]rotaxane **4**^2+^ (125 MHz, CD_2_Cl_2_, 298 K).

Figure S21 - ^1^H-^1^H COSY spectrum of [3]rotaxane **4**^2+^ (500 MHz, CD_2_Cl_2_, 298 K).

Figure S22 - ^1^H-^1^H TOCSY spectrum of [3]rotaxane **4**^2+^ (500 MHz, CD_2_Cl_2_, 298 K).

Figure S23 - ^1^H-^1^H ROESY spectrum of [3]rotaxane **4**^2+^ (500 MHz, CD_2_Cl_2_, 298 K).

Figure S24 - ^1^H -^13^C HSQC spectrum of [3]rotaxane **4**^2+^ (500 MHz, CD_2_Cl_2_, 298 K).

Figure S25 - Partial ^1^H NMR spectra (500 MHz, CD_3_CN, 298 K) of a) [2]rotaxane **6**H^4+^, b) DB24C8, c) [3]rotaxane **1**H^4+^, d) axle **5**H^4+^; color coding of the signals indicates: Bpy^2+^ – blue; Trz^+^ – pink; AmH^+^ – green; DB24C8 – red.

Figure S26 - Partial ^1^H NMR spectra (500 MHz, CD_3_CN, 298 K) of a) [3]rotaxane **1**^3+^, b) [3]rotaxane **3**^3+^, d) [3]rotaxane **4**^2+^; color coding of the signals indicates: Bpy^2+^ – blue; Trz^+^ – pink; AmH^+^ – green; DB24C8 – red.

*Figure S27* - Partial ^1^H NMR spectra (500 MHz, CD_2_Cl_2_, 298 K) of a) [2]rotaxane **6**^3+^, b) [3]rotaxane **1**H^4+^, c) [2]rotaxane **6**H^3+^; color coding of the signals indicates: Bpy^2+^ – blue; Trz^+^ – pink; AmH^+^ – green; DB24C8 – red.

# 4. **^1^H NMR Monitoring of DB24C8 translation to Trz**^+^ **induced by chemical reduction of Bpy**^2+^

The chemical reduction of Bpy^2+^ in compound **3**^3+^ was achieved using cobaltocene (CoCp_2_, *E*_1/2_ = –0.94 V *vs* SCE).^[^^[2]]^ Sample preparation was performed in a glove box under nitrogen atmosphere; to a 1 mM solution of **3**^3+^ in CD_3_CN in a Wilmad low pressure/vacuum NMR tube, were added 2 aliquots of cobaltocene at room temperature. NMR spectra were recorded within 30 minutes after sample preparation. Given the reduction potentials of Bpy^2+^ in **3**^3+^ [*E*_1/2_(Bpy^2+^/Bpy^+·^) = –0.67 V *vs* SCE, *E*_1/2_ (Bpy^+·^/Bpy^0^) = –0.99 V *vs* SCE, see Table S2], after the addition of 2 equivalents of CoCp_2_, the reduction of Bpy^2+^ is quantitative and the majority of the [3]rotaxane is in the radical cation form; in such state, ring B moves from Bpy^.+^ to Trz^+^ as shown by the shift of the protons associated with the triazolium group and adjacent ones (Figure S28b and d). Even if the reduction potential of CoCp_2_ does not allow for the quantitative bi-reduction of Bpy^2+^, an excess of reducing agent can shift the equilibrium of the redox reaction and induce the partial formation of the bi-reduced species. Indeed, after the addition of a large excess of cobaltocene (~ 35 eq.) in solution, a mixture of mono- and bi-reduced rotaxane will be present, with the latter being the most abundant species. In these conditions, the signals associated with the triazolium portion of the molecule undergo further shifts indicating a reorganization of the chemical environment and suggesting that both macrocycles are located at Trz^+^.

Figure S28 - Partial ^1^H NMR spectra (500 MHz, CD_3_CN, 298 K) of a) [3]rotaxane **3**^3+^ (5 mM); b) **3**^3+^ (1 mM) after the addition of 2 eq. of CoCp_2_; c) **3**^3+^ (1 mM) after the addition of 35 eq. of CoCp_2_; d) [2]rotaxane **7**^3+^ (1 mM) after the addition of 10 eq. of CoCp_2_; Dashed lines highlight the variation of chemical shift of relevant signals assigned to Trz^+^ in pink, DB24C8 signals are highlighted in red.

Figure S29 - Partial ^1^H NMR spectra (500 MHz, CD_3_CN, 298 K) of [3]rotaxane **3**^3+^ (1 mM) after the addition of 2 eq. of CoCp_2_. Due to the presence of the radical at Bpy, not all resonances associated with the protons in the molecule are present, the numbers/letters associated with such protons are reported in grey next to the structure of the molecule.

# 5. **p*K*_a_ Determination *via* ^1^H NMR spectroscopy experiments**

The determination of *K*_a_^app^ values of [3]rotaxane **2**H^3+^ and the *K*_a_ value *via* ^1^H NMR titrations was performed by sequential additions of the base quinuclidine (QQ, p*K*_a_ = 19.5)^[^^[3]]^ to a solution of the compound in CD_3_CN at 298K. The concentrations of the species were determined using tetrachloroethane (TCE, Merck) as internal standard. For each spectrum of the titration, *K*_a_^app^ values were calculated considering the concentrations of every species. The final p*K*_a_^app^ value reported corresponds to the average of the p*K*_a_^app^ values calculated excluding the initial 20% and final 20% points of the titration. The reported error was calculated as three times the standard deviation.

Figure S30 - ^1^H NMR stacked spectra (500 MHz, CD_3_CN, 298K) of the deprotonation of **2**H^3+^ ([**2**H^3+^]_i_ = 3.7 mM) after sequential additions of QQ.

Figure S31 - Titration curve showing the variation of concentration of **2**H^3+^ ([**2**H^3+^]_i_ = 3.7 mM) after sequential additions of QQ. Calculated pK_app_ = 18.3 ± 0.1.

# 6. **Relation between apparent acidity constant and co-conformational equilibrium constants of [3]rotaxane 2**H^3+^

*Scheme S2 -* Co-conformational equilibria of **2**H^3+^ for the different states of the ammonium station. The vertical processes represent the deprotonation of AmH^+^, while the horizontal processes represent the shuttling of DB24C8 between AmH^+^ and Bpy^2+^. The counterions of the rotaxane are omitted for sake of clarity. The four species are identified as I, II, III and IV.

Considering Scheme S2, the apparent acidity constant $K_{\text{a}}^{\text{app}}$ of rotaxane **2**H^3+^ is expressed as:

$$K_{\text{a}}^{\text{app}}= \frac{\left( \left[ \text{III} \right]+\left[ \text{IV} \right] \right)\times\text{[H}^{\text{+}}]}{\left[ \text{I} \right]+[\text{II}]} (1)$$

This form takes into account that, due to the shuttling of the ring between AmH^+^ and Bpy^2+^, there are two acidic species (I and II) which can be deprotonated. The acidity constant measured experimentally $K_{\text{a}}^{\text{app}}$ is influenced by the acidity constants of both species ($K_{\text{a}}$ and $K_{\text{a}}^{'}$) and the co-conformational equilibrium constants, which determine the ratio between I and II ($K_{4}$) and III and IV ($K_{5}$).

$K_{7}$ and $K_{8}$ are defined as:

$$K_{7}=\frac{\text{[II]}}{\text{[I]}} (2) K_{8}=\frac{\text{[IV]}}{\text{[III]}} (3)$$

The acidity constants *K*_a_ and *K*_a_^'^ are defined as:

$$K_{\text{a}}=\frac{\text{[III]×}\text{[H}^{\text{+}}\text{]}}{\left[ \text{I} \right]}{(4) K}_{\text{a}}^{'}=\frac{\text{[IV]×}\text{[H}^{\text{+}}\text{]}}{\left[ \text{II} \right]} (5)$$

Combining Equations (1-5), Equation (1) can be rearranged as:

$$K_{\text{a}}^{\text{app}}=\frac{K_{\text{a}}+K_{\text{a}}^{'}\times K_{7}}{1+ K_{7}} (6)$$

And $K_{7}$ can be expressed as:

$$K_{7}=\frac{K_{\text{a}}^{\text{app}}-K_{\text{a}}}{K_{\text{a}}^{'}-K_{\text{a}}^{\text{app}}} (7)$$

While $K_{8}$ is:

$$K_{8}=\frac{K_{\text{a}}^{'}\times K_{7}}{K_{\text{a}}} (8)$$

These relations implies that the values of $K_{7}$ and $K_{8}$ can be obtained from those of $K_{\text{a}}^{\text{app}}$, $K_{\text{a}}$ and $K_{\text{a}}^{'}$.

The value of $K_{\text{a}}$, which corresponds to the acidity constant of AmH^+^ when surrounded by a crown ether ring, can be estimated from the acidity constant of a one-station [2]rotaxane, in which the ring is locked in place. ^[1]^

This relationship is obtained assuming that i) only one ring can surround the ammonium station, ii) both rings can surround the bipyridinium station at the same time; iii) when ammonium unit is deprotonated and the ring moves around Bpy^2+^, the other ring does not move away from the station. All these assumptions are confirmed by NMR data.

*Table S1* - Acidity constants and thermodynamic constants of the co-conformational equilibria in rotaxane **2**H^3+^ (See Scheme S2), in CH_3_CN. ^[a]^ Value taken from reference ^[4]^. ^[b]^ Value taken from reference 1.

| ${\text{p}K}_{\text{a}}$ ^[a]^ | ${\text{p}K}_{\text{a}}^{'}$ ^[b]^ | ${\text{p}K}_{\text{a}}^{\text{app}}$ | $K_{7}$ | $K_{8}$ |
| --- | --- | --- | --- | --- |
| _~_26.3 | 15.2 | 18.3 | 8×10^–4^ | 1×10^8^ |

# 7. **Electrochemical measurements**

Table S2 - Reduction potentials of the investigated species obtained from the Cyclic Voltammetries in CH_3_CN and CH_2_Cl_2_ at room temperature. All potential values are expressed as V vs SCE. ^a^Peak potentials obtained from Differential Pulse Voltammetries.

| **Compound** |  | **CH_3_CN** |  | **CH_2_Cl_2_** | | |  |
| --- | --- | --- | --- | --- | --- | --- | --- |
|  | ***E*_1_**  **(Bpy^2+^/Bpy^+·^)** | ***E*_2_**  **(Bpy^+·^/Bpy^0^)** | ***E*_3_**  **(Trz^+^/Trz^·^)^a^** | ***E*_1_**  **(Bpy^2+^/Bpy^+·^)** | ***E*_2_**  **(Bpy^+·^/Bpy^0^)** | ***E*_3_**  **(Trz^+^/Trz^·^)^a^** | |
| **1**H^4+^ | –0.49 | –0.81 | –1.89 | –0.46 | –0.80 | \ | |
| + P_1_ base (1 eq.) | –0.68 | –0.99 | –2.01 | –0.63 | –0.97 | \ | |
| **2**H^3+^ | –0.53 | –0.99 | \ | –0.50 | –0.99 | \ | |
| + P_1_ base (1.2 eq.) | –0.78 | –1.14 | \ | –0.74 | –1.16 | \ | |
| **3**^3+^ | –0.67 | –0.99 | –2.07 | –0.63 | –0.97 | \ | |
| **4**^2+^ | –0.78 | –1.21 | \ | –0.74 | –1.23 | \ | |

## **7.1 Cyclic Voltammetries and Differential Pulse Voltammetries**

During the cyclic voltammetric experiment, sweeping the potential up to the reduction of the triazolium unit can cause the appearance of extra waves in the anodic scan, due to the poor reversibility of the triazolium reduction process. In such cases two voltammetries are reported, the first stopping before the triazolium reduction and the second one reaching it.

**[3]Rotaxane 1**H^4+^ **in CH_3_CN**

Figure S32 - CV (left, scan rate: 50 mV s^–1^) and DPV (right, scan rate: 20 mV s^–1^, pulse height: 75 mV) of a 2.6×10^–4^ M solution of **1**H^4+^ (black line) and upon sequential addition of P1 base (1 equivalent, red line). Experimental conditions: argon-purged CH_3_CN, room temperature, 100 equivalents of TEAPF_6_.

Two triazolium reductions are observed for **1**H^4+^, as it can be appreciated in the DPV (Fig. S27 left): the first process is related to the protonated [3]rotaxane, while the second process to the deprotonated one. In analogy with the parent [2]rotaxane **6**H^4+^ this behaviour is ascribed to the formation of basic species, upon the reduction of the triazolium, capable to deprotonate the ammonium site of the rotaxane. When the deprotonation process takes place intermolecularly it induces the shuttling of the macrocycle on the triazolium (as the bipyridinium unit is already bireduced), shifting its reduction towards negative potentials and, overall, leading to the presence of two reduction peaks related to this station. The hypothesis was tested repeating the electrochemical measurement of **1**H^4+^ in presence of 0.5 equivalent of triflic acid (*Figure S32*). The addition of triflic acid causes the disappearance of the process at –2.0 V *vs* SCE. Therefore, it can be concluded that the presence of this process is related to a proton transfer triggered by the reduction of the triazolium, which causes the deprotonation of the rotaxane.

Figure S33 - CV (left, scan rate: 100 mV s^–1^) and DPV (right, scan rate: 20 mV s^–1^, pulse height: 75 mV) of a 3.0×10^–4^ M solution of **1**H^4+^ (black line) and upon sequential addition of triflic acid (0.5 equivalent, red line). Experimental conditions: argon-purged CH_3_CN, room temperature, 100 equivalents of TEAPF_6_.

**[3]Rotaxane 1**H^4+^ **in CH_2_Cl_2_**

*Figure S34* - CV (left, scan rate: 100 mV s^–1^) and DPV (right, pulse height: 75 mV) of a 3.1×10^–4^ M solution of **1**H^4+^ (black line, scan rate: 50 mV s^–1^) and upon sequential addition of P1 base (1 equivalent, red line, scan rate: 20 mV s^–1^). Experimental conditions: argon-purged CH_2_Cl_2_, room temperature, 100 equivalents of TBAPF­_6_.

**[3]Rotaxane 2**H^3+^ **in** **CH_3_CN**

Figure S35 - CV (left, scan rate: 200 mV s^–1^) and DPV (right, scan rate: 20 mV s^–1^, pulse height: 75 mV) of a 2.9×10^–4^ M solution of **2**H^3+^ (black line) and upon sequential addition of P1 base (1.2 equivalent, red line). Experimental conditions: argon-purged CH_3_CN, room temperature, 100 equivalents of TEAPF_6_.

**[3]Rotaxane 2**H^3+^ **in CH_2_Cl_2_**

*Figure S36* - CV (left, scan rate: 100 mV s*^–^*^1^) and DPV (right, scan rate: 20 mV s*^–^*^1^, pulse height: 75 mV) of a 2.7×10^–4^ M solution of **2**H^3+^ (black line) and upon sequential addition of P1 base (1.2 equivalent, red line). The marked peak (*) could be related to a degradation product due to the slight excess of P1 base, necessary to fully deprotonate the rotaxane. Experimental conditions: argon-purged CH_2_Cl_2_, room temperature, 100 equivalents of TBAPF­_6_.

**[3]Rotaxane 3**^3+^ **in CH_3_CN**

*Figure S37* - CV (left, scan rate: 100 mV s*^–^*^1^) and DPV (right, scan rate: 20 mV s*^–^*^1^, pulse height: 75 mV) of a 2.8×10*^–^*^4^ M solution of **3**^3+^ (black line). Experimental conditions: argon-purged CH_3_CN, room temperature, 100 equivalents of TEAPF_6_.

*Figure S38* - Stacked plot of 10 CVs (scan rate: 500 mV s*^–^*^1^) of a 2.8×10*^–^*^4^ M solution of **3**^3+^ (scan 1: black line, scans 2-9: gray lines, scan 10: red line), recorded without renewing the diffusion layer. The only difference observed in the CVs is related to a decrease of the background current after the first scan. Experimental conditions: argon-purged CH_3_CN, room temperature, 100 equivalents of TEAPF_6_.

**[3]Rotaxane 3**^3+^ **in CH_2_Cl_2_**

Figure S39 - CV (left, scan rate: 100 mV s^–1^) and DPV (right, scan rate: 20 mV s^–1^, pulse height: 75 mV) of a 2.3×10^–4^ M solution of **3**^3+^ (black line). Experimental conditions: argon-purged CH_2_Cl_2_, room temperature, 100 equivalents of TBAPF_6_.

**[3]Rotaxane 4**^2+^ **in CH_3_CN**

Figure S40 - CV (left, scan rate: 100 mV s^–1^) and DPV (right, scan rate: 20 mV s^–1^, pulse height: 75 mV) of a 3.1×10^–4^ M solution of **4**^2+^ (black line). Experimental conditions: argon-purged CH_3_CN, room temperature, 100 equivalents of TEAPF_6_.

**[3]Rotaxane 4**^2+^ **in CH_2_Cl_2_**

*Figure S41* - CV (left, scan rate: 100 mV s*^–^*^1^) and DPV (right, scan rate: 20 mV s*^–^*^1^, pulse height: 75 mV) of a 4.1×10*^–^*^4^ M solution of **4**^2+^ (black line). Experimental conditions: argon-purged CH_2_Cl_2_, room temperature, 100 equivalents of TBAPF_6_.

## **7.2 Cyclic voltammetries fitting**

Scheme S3 - Co-conformational equilibria of **3**^3+^ for the different redox states of Bpy^2+^. The vertical processes represent the redox reactions of Bpy^2+^, while the horizontal processes represent the shuttling of DB24C8 between Bpy^2+^ and Trz^+^. Counterions are omitted for the sake of clarity.

The cyclic voltammetries of **3**^3+^ were fitted to determine the values of $K_{1}$, $K_{2}$, $K_{3}$, $K_{4}$, $K_{5}$ and $K_{6}$ in CH_3_CN and CH_2_Cl_2_ at 298 K, using the software DigiSim 3.05,^[^^[5]]^ according to the reaction network reported in Scheme S3. CVs at different scan rates ranging from 100 mV s^–1^ to 300 mV s^–1^ were fitted and the equilibrium constant values, obtained from the fitting, were averaged. $K_{1}$ and $K_{2}$ are determined during the fitting, and $K_{3}$, $K_{4}$, $K_{5}$and $K_{6}$ are derived from $K_{1}$ and $K_{2}$ by the following equations:^[^^[6]]^

$$K_{3}=K_{1} e^{{[-F(E_{1}-E_{1}^{'})}/{RT]}} \text{and} K_{4}=K_{2} e^{{[-F(E_{1}^{'}-E_{1}^{''})}/{RT]}}$$

$$K_{5}=K_{3} e^{{[-F(E_{2}-E_{2}^{'})}/{RT]}} \text{and} K_{6}=K_{4} e^{{[-F(E_{2}^{'}-E_{2}^{''})}/{RT]}}$$

Where *F* is the Faraday constant, *R* is the gas constant and *T* is the temperature.

The kinetics of the ring shuttling were considered to be much faster than the scan rate of the CV and the values *k*_1_, *k*_2_ and *k*_3_ were fixed as 10^3^ s^–1^. This consideration is supported by the fact that the reduction processes of Bpy^2+^ are electrochemically reversible in the investigated range of scan rates (50 mV s^–1^ to 1000 mV s^–1^, Figure S41). The reduction potentials of the bipyridinium surrounded by one ($E_{1}^{'}$ and $E_{2}^{'}$) and two rings ($E_{1}$ and $E_{2}$) were taken, respectively, from the model rotaxanes **8**^2+^ (–0.49 V and –1.01 V in CH_2_Cl_2_, –0.55 V and –1.01 V in CH_3_CN)^[1]^ and **4**^2+^ (–0.78 V and –1.21 V in CH_2_Cl_2_, –0.74 V and –1.23 V in CH_3_CN), where the bipyridinium is the only available station for the rings. The reduction potentials $E_{1}^{''}$and $E_{2}^{''}$ of the free bipyridinium were taken from the model axle **5**H^4+^ (–0.25 V and –0.78 V in CH_2_Cl_2_, –0.35 V and –0.79 V in CH_3_CN). The heterogeneous electron transfer constants *k*_et_ of all the processes were fixed as 1 cm s^–1^, value representative of a reversible process under the experimental conditions. The charge-transfer coefficients α were taken as 0.5 in all cases.

*Figure S42* - CV of **3**^3+^ in CH_3_CN (left, 2.3×10^–4^ M) and CH_2_Cl_2_ (right, 2.4×10^–4^ M) at different scan rate: 50 mV s^–1^ (black line), 100 mV s^–1^ (red line), 200 mV s^–1^ (blue line), 300 mV s^–1^ (green line), 500 mV s^–1^ (orange line), 1000 mV s^–1^ (purple line). Experimental conditions: argon-purged CH_3_CN / CH_2_Cl_2_, room temperature, 100 equivalents of TEAPF_6_ / TBAPF_6_.

Figure S42 - Experimental (solid black line) and simulated (dashed red line) CV (scan rate: 100 mV s^–1^) of **3**^3+^ in CH_3_CN (left, 2.8×10^–4^ M) and CH_2_Cl_2_ (right, 2.3×10^–4^ M). Experimental conditions: argon-purged CH_3_CN / CH_2_Cl_2_, room temperature, 100 equivalents of TEAPF_6_ / TBAPF_6_.

*Table S3 -* Thermodynamic constants of the co-conformational equilibria, related to the different oxidation states of the bipyridinium in rotaxane **3**^3+^ (See Scheme S3), obtained by fitting of the cyclic voltammetries in CH_3_CN and CH_2_Cl_2_.

| Solvent | $K_{1}$ | $K_{2}$ | $K_{3}$ | $K_{4}$ | $K_{5}$ | $K_{6}$ |
| --- | --- | --- | --- | --- | --- | --- |
| CH_3_CN | (1.1±0.2)×10^–2^ | (1.3±0.4)×10^–7^ | (9±2)×10^1^ | (3.2±0.9)×10^–4^ | (1.4±0.3)×10^4^ | 1.7±0.5 |
| CH_2_Cl_2_ | (5±1)×10^–3^ | (5±1)×10^–8^ | (8±2)×10^1^ | (6±1)×10^–4^ | (2.8±0.6)×10^4^ | 4.7±0.9 |

## **7.3 Relation between reduction potential of Trz**^+^ **and co-conformation equilibrium constants of [3]rotaxane 1**^3+^

The difference in $E_{3}$ values of **1**^3+^ and **3**^3+^ (–2.01 and –2.07 V *vs* SCE, respectively) can be ascribed to the presence of the amine site in **1**^3+^ instead of the acetylated amine of **3**^3+^. This unit can act as an extremely weak station for the ring,^[^^[7]]^ and, when Bpy is doubly reduced and Trz^+^ is already surrounded by one ring, it could affect the co-conformational equilibria of the rotaxane. It must be emphasized that, in the following, the reduction potentials of Trz^+^ will be estimated from the DPV peak potentials recorded at a very slow scan rate. This is an approximation because the Trz^+^ reduction is poorly reversible; therefore, the following discussion must be kept at a qualitative level.

*Scheme S4 -* Co-conformational equilibria of **1**^3+^ (after the two-electron reduction of Bpy) for the different states of the triazolium station. The vertical processes represent the redox reactions of Trz^+^, while the horizontal processes represent the shuttling of DB24C8 between Trz^+^ and Am. The counterions of the rotaxane are omitted for sake of clarity. The four species are identified as I, II, III and IV.

Considering Scheme S4, the apparent reduction potential $E_{3}$ of Trz^+^ in rotaxane **1**^3+^ (after the two-electron reduction of Bpy) may be expressed by the Nernst equation at equilibrium,^[^^[8]]^ under the assumption – consistent with the voltametric observations – that the co-conformational reactions are much faster than the electrode potential scan rate:

$0=E_{3}-\frac{RT}{F}\text{ln}K_{\text{r}}^{\text{app}} (9)$ $K_{\text{r}}^{\text{app}}=\frac{{\text{(}\left[ \text{III} \right]\text{+}\left[ \text{IV} \right]\text{)}}_{\text{eq}}}{{\text{(}\left[ \text{I} \right]\text{+}\left[ \text{II} \right]\text{)}}_{\text{eq}}} (10)$

where *F* is the faraday constant, *R* is the gas constant, *T* is the temperature, and $K_{\text{r}}^{\text{app}}$ is the apparent equilibrium constant of the redox reaction involving the oxidized species I and II and the reduced species III and IV. Thus, eq. 11 may be obtained.

$$E_{3}=\frac{RT}{F}\text{ln}\frac{\text{[III]+[IV]}}{\text{[I]+[II]}}\text{ }\text{ }(11)$$

This treatment considers that, due to the shuttling of ring A between Trz^+^ and Am, there are two species that can be reduced (I and II). The reduction potential measured experimentally $E_{3}$ is influenced by the reduction potentials of both species ($E_{3}^{'}$ and $E_{3}^{''}$) and the co-conformational equilibrium constants, which determine the ratio between I and II ($K_{9}$) and III and IV ($K_{10}$). If the kinetics of ring shuttling are faster than the voltammetry scan rate, the shuttling will re-adjust the concentrations of the redox active species during the voltametric experiment.

$K_{9}$ and $K_{10}$ are defined as:

$$K_{9}=\frac{\text{[II]}}{\text{[I]}} (12) K_{10}=\frac{\text{[IV]}}{\text{[III]}} (13)$$

The reduction potentials $E_{3}^{'}$ and $E_{3}^{''}$ can be expressed as:

$$E_{3}^{'}=\frac{RT}{F}\text{ln}\frac{\text{[III]}}{\text{[I]}} (14) E_{3}^{''}=\frac{RT}{F}\text{ln}\frac{\text{[IV]}}{\text{[II]}} (15)$$

Combining Equations (11-15), $K_{9}$ can be expressed as:

$$K_{9}=\frac{e^{\frac{F}{RT}E_{3}^{''}}-e^{\frac{F}{RT}E_{3}}}{e^{\frac{F}{RT}E_{3}}-e^{\frac{F}{RT}E_{3}^{'}}} (16)$$

While $K_{10}$ is:

$$K_{10}=\frac{e^{\frac{F}{RT}E_{3}^{''}}}{e^{\frac{F}{RT}E_{3}^{'}}}K_{9} (17)$$

These relations implies that the values of $K_{9}$ and $K_{10}$ can be obtained from those of $E_{3}$, $E_{3}^{'}$ and $E_{3}^{''}$.

The value of *E*_3_ can be approximated to the peak potential of Trz^+^ in **1**^3+^ taken from DPV (–2.01 V *vs* SCE). The value of $E_{3}^{'}$ can be approximated to the peak potential of Trz^+^ in **3**^3+^ taken from DPV (–2.07 V *vs* SCE), in which two rings surround Trz^+^ after the reduction of Bpy^2+^. The value of $E_{3}^{''}$ can be approximated to the potential of Trz^+^ in **8**^3+^ taken from DPV (–1.91 V *vs* SCE), in which one ring surrounds Trz^+^ after the reduction of Bpy^2+^. The values estimated for $K_{9}$ and $K_{10}$ are reported in Table S4.

*Table S4 -* Thermodynamic constants of the co-conformational equilibria in doubly reduced rotaxane **1**^3+^ in CH_3_CN (See Scheme S4).

| $K_{9}$ | $K_{10}$ |
| --- | --- |
| 0.02 | 10 |

# **References**

1. []	L. Andreoni, J. Groppi, O. Seven, M. Baroncini, S. Silvi, A. Credi, *Angew. Chem. Int. Ed.* **2025**, *64*, e202414609
2. []	W. E. Geiger, *J. Am. Chem. Soc.* **1974**, *96*, 2632-2634.
3. []	Z. Glasovac, M. Eckert-Maksic, Z. Maksic, *New J. Chem.* **2009,** 33, 588-597.
4. []	M. J. Power, D. T. J. Morris, I. J. Vitorica-Yrezabal, D. A. Leigh, *J. Am. Chem. Soc.* **2023**, 145, 15, 8593-8599.
5. []	[www.basinc.com/products/ec/digisim](http://www.basinc.com/products/ec/digisim)
6. [] A. Kaifer, M. Gómez-Kaifer, *Supramolecular Electrochemistry*, Wiley‐VCH, Weinheim, **1999**.
7. []	B. Riss-Yaw, J. Morin, C. Clavel, F. Coutrot, *Molecules* **2017,** *22*, 2017.
8. [] A. J. Bard, L. R. Faulkner, *Electrochemical Methods: Fundamentals and Applications*, John Wiley & Sons Inc., New York, **2001**
